# Predicting mortality in the intensive care unit: a comparison of the University Health Consortium expected probability of mortality and the Mortality Prediction Model III

**DOI:** 10.1186/s40560-016-0158-z

**Published:** 2016-05-23

**Authors:** Angela K. M. Lipshutz, John R. Feiner, Barbara Grimes, Michael A. Gropper

**Affiliations:** Department of Anesthesia and Perioperative Care, University of California, 521 Parnassus Avenue, San Francisco, CA 94143 USA; Department of Epidemiology and Biostatistics, University of California, 185 Berry Street, Lobby 5, Suite 5700, San Francisco, CA 94107 USA

**Keywords:** Critical care, Intensive care, Mortality, Severity of illness index, Projections and predictions

## Abstract

**Background:**

Quality benchmarks are increasingly being used to compare the delivery of healthcare, and may affect reimbursement in the future. The University Health Consortium (UHC) expected probability of mortality (EPM) is one such quality benchmark. Although the UHC EPM is used to compare quality across UHC members, it has not been prospectively validated in the critically ill. We aimed to define the performance characteristics of the UHC EPM in the critically ill and compare its ability to predict mortality with the Mortality Prediction Model III (MPM-III).

**Methods:**

The first 100 consecutive adult patients discharged from the hospital (including deaths) each quarter from January 1, 2009 until September 30, 2011 that had an intensive care unit (ICU) stay were included. We assessed model discrimination, calibration, and overall performance, and compared the two models using Bland-Altman plots.

**Results:**

Eight hundred ninety-one patients were included. Both the UHC EPM and the MPM-III had excellent performance (Brier score 0.05 and 0.06, respectively). The area under the curve was good for both models (UHC 0.90, MPM-III 0.87, *p* = 0.28). Goodness of fit was statistically significant for both models (UHC *p* = 0.002, MPM-III *p* = 0.0003), but improved with logit transformation (UHC *p* = 0.41; MPM-III *p* = 0.07). The Bland-Altman plot showed good agreement at extremes of mortality, but agreement diverged as mortality approached 50 %.

**Conclusions:**

The UHC EPM exhibited excellent overall performance, calibration, and discrimination, and performed similarly to the MPM-III. Correlation between the two models was poor due to divergence when mortality was maximally uncertain.

## Background

Quality benchmarking is increasingly being used to evaluate the delivery of healthcare on the individual and systems levels by comparing performance measures among providers and hospitals. The goal of benchmarking is to improve transparency in healthcare and highlight methods that can be utilized to improve quality [1, 2]. Pay-for-performance (P4P) programs, such as those mandated by the Affordable Care Act (http://www.healthcare.gov/law/full/index.html, accessed November 18, 2014), utilize quality benchmarking by tying financial incentives to performance [3]. However, the integrity of quality benchmarking depends on fair comparison of the performance measures being utilized.

Performance measures can be either process or outcomes measures. Process measures examine compliance with a task, such as administration of deep vein thrombosis prophylaxis in the critically ill or administration of aspirin in a patient having a myocardial infarction. Outcome measures evaluate the result of the care provided; such measures include hospital mortality or functional independence after discharge from the intensive care unit (ICU). Comparison of outcomes measures among different providers and hospital systems requires case-mix adjustment, so that individuals and systems are not penalized for taking care of sicker patients. Process measures were initially thought to be unaffected by severity of illness, negating the need for case-mix adjustment for comparison; however, this assumption has recently been called into question (http://gold.ahrq.gov/projectsearch/grant_summary.jsp?grant=R01+HS18338-01A1, accessed November 18, 2014). Thus, adequate case-mix adjustment is of paramount importance when comparing performance measures across providers and hospitals.

Case-mix adjustment is typically accomplished utilizing prognostic models that predict a patient’s probability of mortality. To this end, prognostic models have been calibrated and validated in various hospital populations. However, each model relies on a different set of variables to predict mortality and, as such, may perform differently in different populations. The Acute Physiology and Chronic Health Evaluation (APACHE) [4], Simplified Acute Physiology Score (SAPS) [5–7], and Mortality Probability Model (MPM) [8, 9] were created explicitly for use in the ICU, validated in the critically ill, and rely primarily on physiologic data to predict mortality. The University Health Consortium (UHC) expected probability of mortality (EPM) is another model that can be used to predict mortality in hospitalized patients. The UHC EPM differs from the APACHE, SOFA, and MPM in that it was not created explicitly for use in the ICU. Furthermore, it relies on administrative data, calculated based on a complex algorithm that includes diagnosis-related group (DRG) and comorbidities (UHC, Risk Adjustment Methodology for the Clinical Data Base, Oak Brook, IL, 2011). The benefit of using administrative data over physiologic data is that it is easier and less expensive to collect; however, it depends on the accuracy of documentation by providers. Although the UHC EPM was internally calibrated and validated, its prognostic ability in certain populations has been questioned [10, 11]. It has not been extensively studied in the critically ill. To date, only one study has evaluated the use of the UHC EPM specifically in the ICU: Enfield et al. compared the performance of the UHC EPM and the APACHE-IV in critically ill patients at two academic medical centers and found that the UHC EPM was adversely affected by severity of illness [12]. However, the study was limited to medical ICU patients, and the sample size was relatively small.

At the University of California, San Francisco, we use the UHC EPM to aid in benchmarking our critical care services against other UHC member institutions. Thus, we sought to define the performance characteristics of the UHC EPM in our ICU population and to compare its ability to predict mortality with the MPM-III.

## Methods

### Prognostic models

#### UHC EPM

UHC is an alliance of 119 academic medical centers and their 293 hospital affiliates, representing approximately 90 % of US non-profit academic medical centers (https://www.uhc.edu/cps/rde/xchg/wwwuhc/hs.xsl/home.htm, accessed April 13, 2013). Member hospitals provide de-identified patient level data to UHC for inclusion in the UHC Clinical Data Base; UHC then applies a proprietary model based on DRG and comorbidities to calculate the EPM (UHC, Risk Adjustment Methodology for the Clinical Data Base, Oak Brook, IL, 2011). The EPM model was internally calibrated and internally validated. It is estimated to predict 84 % of the odds of death in critical illness [12]. The University of California, San Francisco (UCSF) Medical Center is a UHC member and provides patient level data coded from clinical documentation to the Clinical Data Base for quality improvement purposes.

#### MPM-III

The MPM-III estimates hospital mortality based on 16 variables collected within 1 h of ICU admission [9]. The MPM-III is an update of the MPM-II; variables included in the MPM-III were identified via a retrospective analysis of 124,855 Project IMPACT (Cerner Project IMPACT, Inc., Bel Air, MD) patients. The variables featured in the model include physiologic parameters, and acute and chronic diagnoses, such as heart rate, systolic blood pressure, acute renal failure, history of chronic kidney disease, history of cirrhosis, and history of malignant neoplasm. The MPM-III is well-calibrated and shows excellent discrimination in the Project IMPACT sample.

### Setting and subjects

The UCSF Medical Center is a 560-bed tertiary care academic medical center in San Francisco, CA. The medical center has 77 adult critical care beds (32 medical/surgical, 29 neurology/neurosurgical, and 16 cardiology/cardiothoracic surgical) in a semi-closed ICU setting with mandatory intensivist consultation for all medical/surgical patients as well as neurology/neurosurgical and cardiology/cardiothoracic surgical patients requiring mechanical ventilation.

We included the first 100 consecutive adult patients discharged from the hospital (including deaths) each quarter between January 1, 2009, and September 30, 2011, who required an ICU stay. We obtained data, including the EPM, from the UHC Clinical Data Base directly from UHC for the entire study period. Data to calculate MPM-III for the first 100 consecutive discharges per quarter during the study period was obtained via retrospective review of the clinical record as part of our institution’s involvement in the California Hospital Assessment and Reporting Taskforce (http://www.calqualitycare.org/about, accessed November 18, 2014). The UHC and MPM-III data were merged by patient encounter number, a unique identifier for each patient and hospitalization. Repeat admissions, trauma patients, burn patients, patients admitted to rule-out myocardial infarction who were subsequently ruled out (and had no other indications for ICU admission), and patients admitted immediately after coronary artery bypass grafting were excluded. Additionally, patients without complete information available with which to calculate the MPM-III score were excluded.

### Statistical analysis

Approval for this study was obtained from the UCSF Institutional Review Board, and the requirement for written informed consent was waived. Descriptive data were summarized as number (percentage), mean ± standard deviation (SD), mean (95 % confidence interval (CI)), or median (interquartile range (IQR)). Predicted mortalities were compared using the paired *t* test and Wilcoxon sign rank test. Overall model performance was assessed via Brier scores, which measure the average squared deviation between the predicted probabilities for a set of events and their outcomes. A lower score indicates a more accurate prediction [13]. Each model’s discriminative ability was assessed via its receiver operator characteristic (ROC) curve. Model calibration was described via the Hosmer-Lemeshow goodness-of-fit test. The mortality index for each model was calculated using observed and expected mortality.

The UHC EPM and MPM-III were compared using Pearson’s correlation coefficient, Spearman rho, and Bland-Altman plots. Given poor calibration in the original models, we performed a post hoc analysis using logit-transformed models to assess for improved model fit. We hypothesized that, since one model relies on administrative data and the other on clinical data, the use of both models together would improve the prediction of mortality. To test this hypothesis, we performed multivariate logistic regression for the outcome of hospital mortality with each logit-transformed model as a predictor. We checked for interaction between the two models; since there was a statistically significant interaction, the interaction term was included in the final model. We then compared this model to the univariate logistic regression results for each model separately using the likelihood ratio test. We attempted to identify MPM-III model variables that, when added individually to the logit-transformed UHC model, improved its predictive ability. We chose the UHC EPM as the base model for this analysis since the UHC EPM is based on administrative data that is already collected for coding and billing, rendering its calculation easy and inexpensive. While collection of the physiologic data for the MPM-III is tedious, time-consuming, and costly even in the era of electronic medical records, collecting one variable could be worthwhile if it improves the predictive capability of the model significantly. This analysis was performed by comparing the area under the ROC curves for the UHC EPM plus individual MPM-III variables and the UHC EPM alone, as well as calculating net reclassification indices. The net reclassification index is a measure for evaluating the improvement in predictive performance obtained by adding a marker to a baseline set of predictors—in this case, adding individual MPM-III variables to the UHC EPM predictors. Reclassification is considered separately for those who experience events and those who do not. Reclassification to a risk group with a higher risk is an upward movement and is an improvement in classification for those experiencing an event, while reclassification downward is a failure for those who have an event. For those who do not have an event, reclassification downward is an improvement, while reclassification upward is a failure. The reclassification index is calculated by summing the proportions of subjects reclassified upward minus those reclassified downward for those having an event and those reclassified downward minus those reclassified upward for those not having an event [14]. For our analysis, we used mortality cut points of <25, 25–50, and >75 % to represent low, moderate, and high predicted mortality, respectively.

Additionally, since the models may perform differently in different ICU populations, we performed a pre-planned subgroup analysis to evaluate model performance in the medical-surgical ICU population (excluding cardiac/cardiothoracic and neurologic/neurosurgical ICU patients). Statistical analysis was performed using Stata/IC 12.1 (StataCorp, College Station, TX).

## Results

**Fig. 1:**
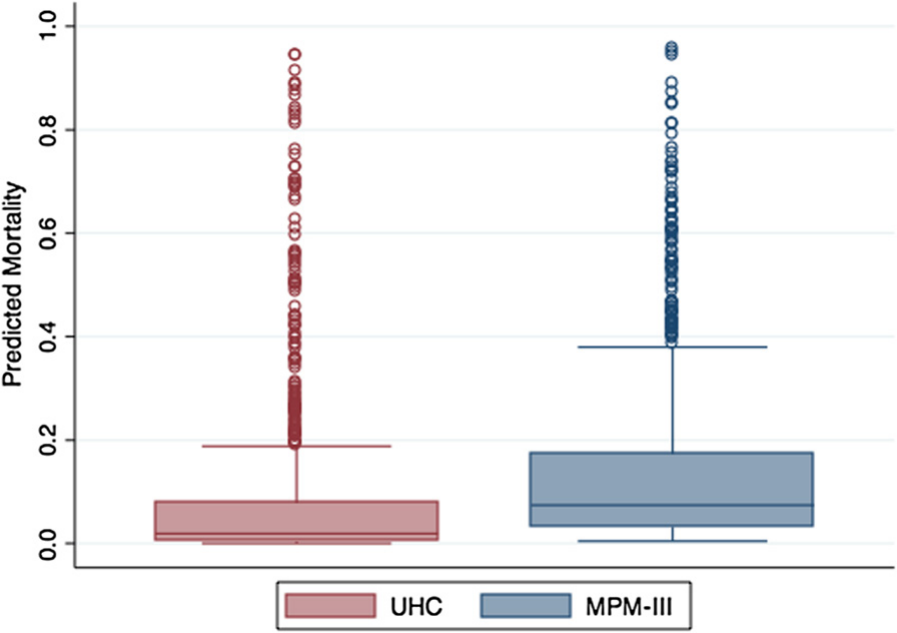
Box and whisker plots of predicted mortality for the University Health Consortium (UHC, *red*) and Mortality Probability Model III (MPM-III, *blue*) are shown. The *box* shows the median and interquartile range (IQR). The *whiskers* display the upper and lower values within 1.5 times the IQR beyond the 25th and 75th percentiles. Individual data points represent outliers. The median (IQR) predicted mortality was 1.90 % (0.36–7.78) for the UHC model and 7.40 % (3.21–17.34) for the MPM-III, (Wilcoxon sign rank *p* < 0.0001)

A total of 891 patients were included in the analysis, of which 65 (7.3 %) died. Patient demographics are described in Table 1. The UHC and MPM-III mean predicted mortalities were 8.22 % (95 % CI 7.17–9.28) and 14.29 % (95 % CI 13.16–15.44), respectively (paired *t* test *p* < 0.0001); median predicted mortalities were 1.90 % (IQR 0.36–7.78) and 7.40 % (IQR 3.21–17.34), respectively (Wilcoxon sign rank *p* < 0.0001) (Fig. 1). The average ratio of observed to expected mortality was 0.83 for the UHC model and 0.52 for the MPM-III model.

**Table 1:** Patient characteristics

Number	891
Age	
Survivors	55.6 ± 17.1
Non-survivors	61.0 ± 17.5
Gender	
Male	471 (52.9 %)
Female	420 (47.1 %)
Ethnicity	
White	507 (56.9 %)
Black	73 (8.2 %)
Asian	46 (5.2 %)
Hispanic	57 (6.4 %)
Other	208 (23.3 %)
Admission type	
Medical	204 (22.9 %)
Surgical	251 (28.2 %)
Cardiac/cardiothoracic	139 (15.6 %)
Neurological/neurosurgical	292 (32.8 %)
Unknown	5 (0.6 %)
DNR within 24 h of admission	29 (3.3 %)
Duration of mechanical ventilation	2 (1-5)
ICU length of stay in days	2 (1-6)
Hospital length of stay in days	8 (4-14)
Hospital discharge destination: discharged home	440 (49.4 %)
Hospital mortality	65 (7.3 %)

Data are mean ± SD, *n* (%), or median (interquartile range)

*DNR* do not resuscitate, *ICU* intensive care unit

**Fig. 2:**
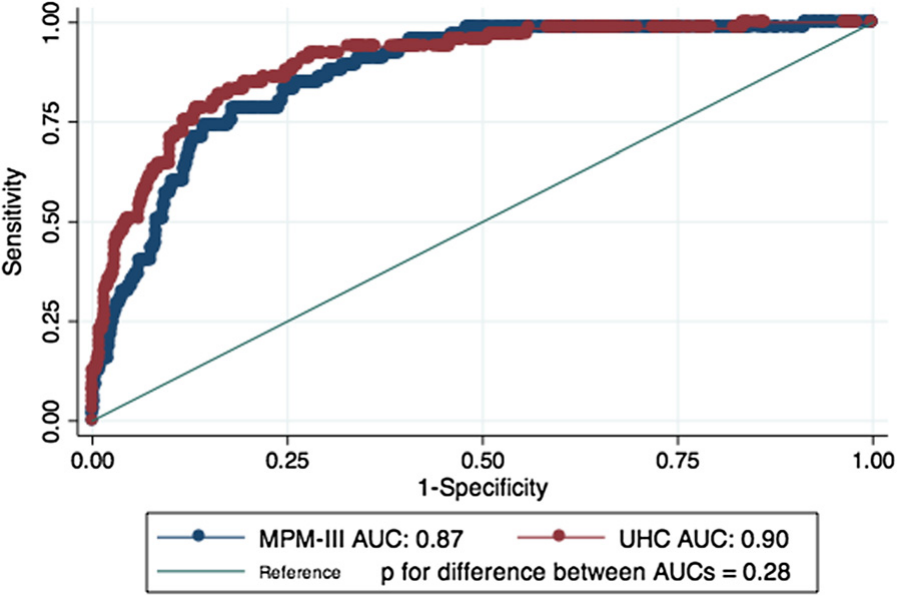
The receiver operating characteristic curves (ROC) plotting sensitivity vs. 1-specificity are shown separately for the University Health Consortium (UHC, *red*) and Mortality Probability Model III (MPM-III, *blue*). The area under the curve (AUC) for the UHC curve was 0.90 (95 % CI 0.86–0.93). The AUC for the MPM-III was 0.87 (95 % CI 0.83–0.91). The curves were not statistically different (*p* = 0.28). The *diagonal line* shows where there is no discriminating ability (AUC = 0.5)

**Fig. 3:**
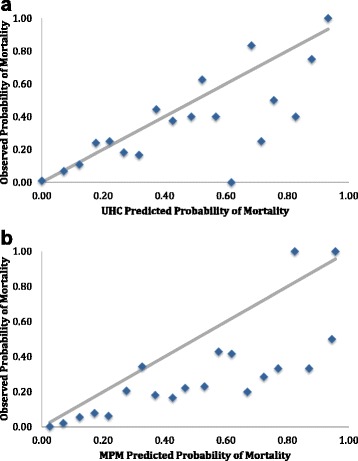
The predicted probability of mortality is plotted against the observed probability of mortality for the University Health Consortium (UHC) in panel **a** and Mortality Probability Model III (MPM) in panel **b**. The *diagonal line* shows where predicted and observed probabilities of mortality are equal

Both the UHC model and the MPM-III had excellent overall performance (Brier score 0.05 and 0.06, respectively). The ROC curves (Fig. 2) showed excellent discrimination for both models (area under the curve, UHC 0.90, 95 % CI 0.86–0.93; MPM-III 0.87, 95 % CI 0.83–0.91; *p* for difference = 0.28). The Hosmer-Lemeshow goodness-of-fit test was statistically significant for both models (UHC *Χ*^2^ = 24.09, *p* = 0.002; MPM-III *Χ*^2^ = 28.93, *p* = 0.0003), suggesting a poor model fit. Logit transformation of the models improved goodness of fit (UHC *Χ*^2^ = 8.29, *p* = 0.41; MPM-III *Χ*^2^ = 14.54, *p* = 0.07). Calibration plots suggest that the MPM-III overestimates mortality (Fig. 3).

**Fig. 4:**
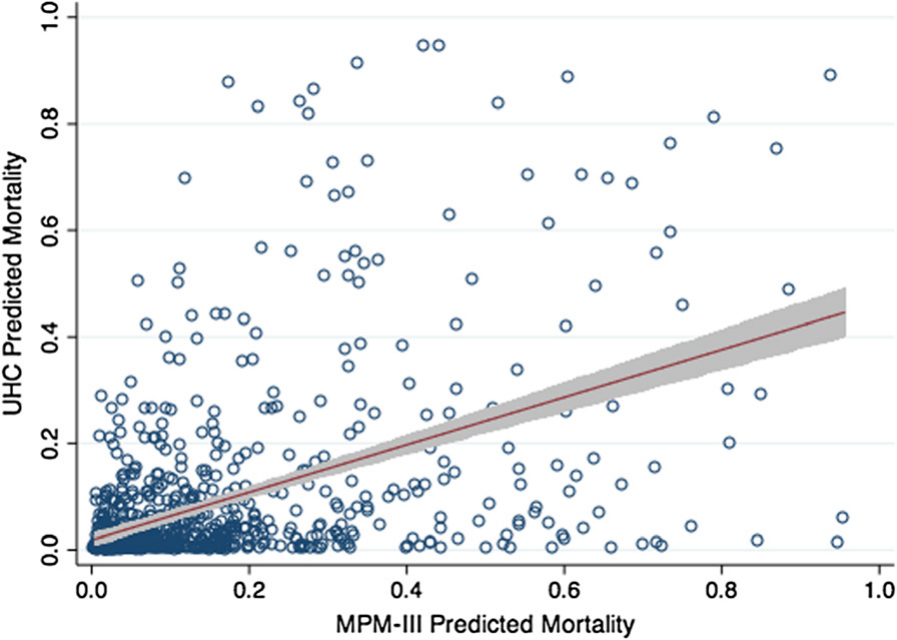
Mortality Probability Model III (MPM-III) predicted mortality is plotted against the University Health Consortium (UHC) predicted mortality. Each data point represents a single patient. The *line* represents the regression line, with the *shaded area* representing the 95 % confidence interval. The Pearson correlation coefficient was 0.48, while the Spearman rho was 0.50, both *p* < 0.0001

The two models correlated weakly, with a Pearson correlation coefficient of 0.48 (*p* < 0.0001) and Spearman rho of 0.50 (*p* < 0.0001) (Fig. 4).

**Fig. 5:**
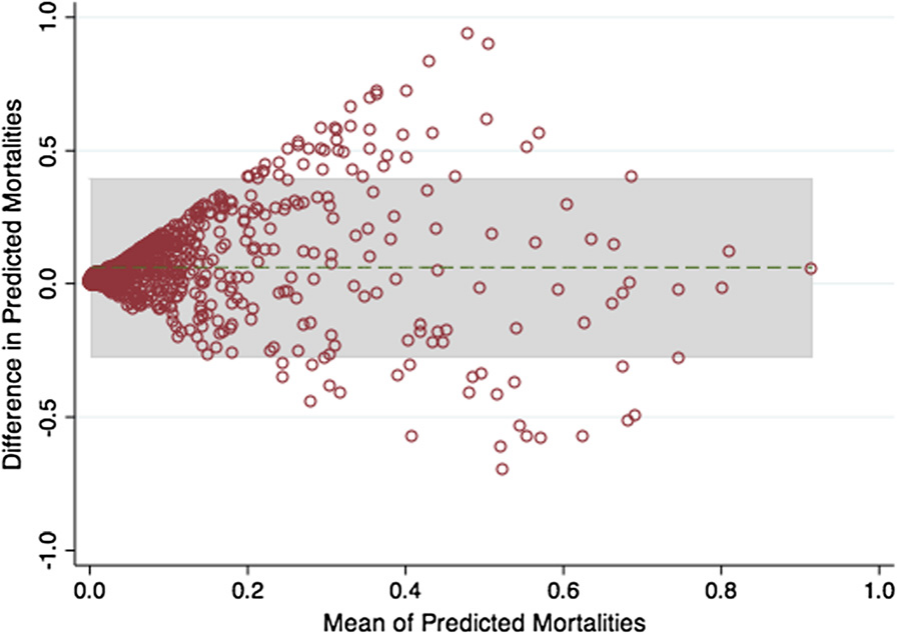
The Bland-Altman plot shows the difference between the University Health Consortium (UHC) and Mortality Probability Model III (MPM-III) plotted against the average predicted mortalities. The *green horizontal dashed line* represents the overall mean of the differences, while the *shaded area* shows the limits of agreement

The Bland-Altman plot (Fig. 5) shows good agreement between the two models at extremes of mortality but poor agreement when mortality is maximally uncertain (i.e., as predicted mortality approaches 50 %).

Multivariate logistic regression with the logit-transformed UHC model and the logit-transformed MPM-III as predictor variables revealed that each model was independently predictive of the log odds of death (beta coefficient 0.66 (95 % CI 0.47–0.84) and 0.64 (95 % CI 0.39–0.89) for the UHC and MPM-III models, respectively). However, when an interaction term was included in the analysis, it was statistically significant (*p* = 0.005), suggesting that the effect of each predictor depended on the value of the other. Likelihood ratio testing revealed that use of the UHC EPM and MPM-III together improved the predictive capability as compared to each model alone (likelihood ratio *Χ*^2^ for combined model vs. UHC EPM alone = 35.44, *p* < 0.0001; likelihood ratio *Χ*^2^ for combined model vs. MPM-III alone = 75.78, *p* < 0.0001).

Post hoc analysis evaluating the effect of adding individual variables from the MPM-III to the logit-transformed UHC model on the area under the ROC curve, and net reclassification index identified four individual variables that, when added to the logit-transformed UHC model, resulted in improved prognostic abilities. Of the four variables—coma (defined as Glasgow Coma Score of 3 or 4) at the time of ICU admission, cardiopulmonary resuscitation within 24 h prior to ICU admission, mechanical ventilation within 1 h of ICU admission, and limitations on emergency therapies or interventions (e.g., do not resuscitate or do not intubate orders) present at the time of ICU admission—only limitations on emergency therapies reached statistical significance for both the area under the curve and net reclassification index (Table 2).

**Table 2:** Area under the receiver operating characteristic curve and net reclassification index for University Health Consortium (UHC) Model plus individual Mortality Prediction Model III (MPM-III) variables

MPM-III variables	AUC for UHC model + additional variable	*p* value for difference between AUCs	NRI	NRI *p* value
Coma	0.91	0.09	0.09	0.04
Heart rate >150	0.90	0.36	0.03	0.14
Systolic blood pressure <90	0.90	0.34	0.00	N/A
Chronic kidney disease	0.90	0.49	0.00	N/A
Cirrhosis	0.90	0.90	0.02	0.29
Malignant neoplasm	0.90	0.74	0.00	N/A
Acute renal failure	0.90	0.48	0.01	0.36
Arrhythmia	0.90	0.32	0.00	0.87
Cerebrovascular accident	0.90	0.92	0.02	0.29
Gastrointestinal bleed	0.90	0.44	0.00	0.56
Intracranial mass effect	0.90	0.72	0.00	0.16
CPR prior to admission	0.90	0.14	0.12	0.01
Mechanical ventilation within 1 h of admission	0.91	0.03	0.04	0.50
Medical or unscheduled surgical admission	0.90	0.22	−0.01	0.78
Limitation on emergency therapy or intervention	0.92	0.03	0.10	0.05
All MPM-III variables	0.92	0.01	N/A	N/A

Net reclassification index represents the proportion of patients who were appropriately recategorized into low (<25 %), moderate (25–50 %), and high (>75 %) risk of mortality with the addition of each individual MPM-III variable

*AUC* area under curve, *CPR* cardiopulmonary resuscitation, *MPM* Mortality Probability Model III, *NRI* net reclassification index, *UHC* University Health Consortium

Since the models may perform differently in different patient populations, we performed a pre-planned subgroup analysis of model performance in the medical-surgical ICU patients, excluding neurological/neurosurgical ICU patients and cardiac/cardiothoracic surgical ICU patients. A total of 460 patients were included in this subgroup analysis. The mean age was 57.0 (SD, 17.8) years, 246 (53 %) patients were male, and 234 (51 %) were white. Median hospital and ICU LOS were 9 (IQR, 5–17) and 3 (IQR, 1–6) days, respectively. Median duration of mechanical ventilation was 3 (IQR, 2–6) days. Hospital mortality in this subgroup was 9.13 %. Median predicted mortality was 2.40 % (IQR 0.41–11.18) for the UHC model and 9.69 % (IQR 3.95–21.15) for the MPM-III (Wilcoxon sign rank *p* < 0.001). Brier score was 0.07 and 0.08 for the UHC EPM and MPM-III, respectively. ROC curves were similar for the two models (area under the curve, UHC 0.88, 95 % CI 0.84-0.93; MPM-III 0.85, 95 % CI 0.80-0.91, *p* for difference 0.44). The Hosmer-Lemeshow goodness-of-fit test was statistically significant for the UHC model (*Χ*^2^ = 20.61, *p* = 0.01) but not the MPM-III (*Χ*^2^ = 10.04, *p* = 0.26).

## Discussion

Our study sought to define the performance characteristics of the UHC EPM in our ICU population, and compare the ability of the UHC EPM and MPM-III to predict death in our heterogeneous cohort of adult ICU patients. In this cohort, which included medical/surgical, cardiology/cardiothoracic surgery, and neurology/neurosurgical ICU patients, the UHC EPM exhibited excellent overall performance, discrimination, and calibration in predicting ICU mortality, suggesting that although it was not designed specifically for use in ICU patients nor prospectively validated in the critically ill, it is able to predict mortality in this population reasonably well. Additionally, the UHC EPM exhibited similar discrimination, as depicted by the ROC curves, to the MPM-III, a model created and validated for the prediction of mortality in the critically ill.

However, the two models differed in several important ways. First, the MPM-III consistently overestimated mortality in our population. The UHC EPM did not overestimate mortality, potentially making it a better model for quality benchmarking across ICUs, since overestimation of mortality may overstate the quality of care provided in poorly performing ICUs. Additionally, correlation between the two models was poor due to divergence of the models when mortality was maximally uncertain (i.e., when predicted mortality approached 50 %). Thus, ICUs with moderately ill patients may perform quite differently depending on which model is used, and appropriate model selection may depend on the patient population and severity of illness.

Likelihood ratio testing confirmed that combining the two models provided superior predictive capabilities as compared to either model alone. This finding is likely due to the fact that the two models rely on different types of information—the UHC EPM relies on primarily administrative data, while the MPM-III relies on physiologic data and comorbidities. Several MPM-III variables, when added individually to the logit-transformed UHC model, improve the predictive ability of the model. Since collection of a single variable may not be overly time-consuming or expensive, the addition of a single physiologic variable to the administrative model should be considered.

Literature on the performance of the UHC model is scarce. Davenport et al. compared the UHC Clinical Data Base to the National Surgical Quality Improvement Program (NSQIP) database in a cohort of over 26,000 surgical patients and found that the NSQIP database was superior at predicting death and complications [10]. Kozower et al. compared the UHC mortality risk score to that of the Society of Thoracic Surgeons (STS) in a cohort of cardiac surgical patients and found that the preoperative risk predicted by the UHC model was influenced by postoperative complications [11]. However, neither of these studies looked specifically at the use of the UHC model in the ICU. More recently, Enfield et al. compared the performance UHC EPM and APACHE-IV in 556 medical ICU patients at two institutions and found that although both models had outstanding discrimination, the goodness-of-fit tests were statistically significant, with divergence as predicted mortality increased [12]. Additionally, the administrative data was influenced by severity of illness, and the two models differed in their conclusions when comparing the two institutions. Thus, the authors cautioned against relying on administrative data for comparisons of quality among hospitals.

Our study differs from the previous studies in several ways. First, unlike the studies of Davenport et al. and Kozower et al., our study focused entirely on the ICU population. And unlike the study by Enfield et al., we included surgical ICU patients, cardiology/cardiothoracic surgical ICU patients, and patients from the neuroscience ICU. Additionally, our sample size was larger than that of Enfield et al., giving us more power to detect differences between the two models. In our study, divergence was most profound when mortality was maximally uncertain, and goodness of fit testing was not statistically significant when using logit-transformed models, suggesting good model fit. Furthermore, in our analysis, the physiologic model consistently overestimated mortality. Therefore, the administrative model appears to perform well in our ICU population. Despite these differences, our findings do agree with the conclusion by Enfield et al. that “the methodology used to develop a mortality prediction model can influence how these models compare [and]…two models can have similar AUC and still perform remarkably differently.”

Our study has several limitations. First, our analysis includes only the first 100 consecutive discharges per quarter who required intensive care. Although the patient characteristics of this subgroup were similar to that of all patients discharged who required ICU level care over the study period (data not shown), there could be unrecognized differences that affect our results. Second, while our sample size is larger than previous studies of the UHC model in ICU patients [12], it is small compared to validation cohorts used to assess the performance of the APACHE [4], SAPS [5–7], and MPM [8, 9]. Third, although the inclusion of subspecialty ICU patients, such as cardiology/cardiac surgery and neurology/neurosurgery patients, improves the generalizability of our study, the heterogeneity of our ICU population may affect our results if the models perform differently in different patient subsets. Of note, however, the two models performed similarly in a pre-planned subgroup analysis of only medical/surgical ICU. Fourth, our attempt to identify individual variables from the MPM-III model that, when added to the UHC EPM, improved model performance based on the net reclassification index depended largely on the cut points used. We chose <25, 25–50, and >75 % to represent low, moderate, and high predicted mortality, respectively. The use of alternate cut points may result in different results. Additionally, as with all administrative data, the integrity UHC data relies on the quality of coding; this documentation was not reviewed to ascertain the quality of data that drives this model. Poor coding could lead to under- or overestimation of mortality risk. Furthermore, since the UHC EPM is a proprietary algorithm, institutions that are not UHC members are unable to calculate their predicted mortality using this method. And, finally, since this study was performed at a single academic medical center, our results may not be generalizable to other settings.

## Conclusions

The UHC EPM exhibited excellent overall performance, calibration, and discrimination in our ICU population, and performed similarly to the MPM-III model. However, correlation between the two models was poor due to divergence of the models when mortality was maximally uncertain. Even when two models have similar characteristics, they can perform quite differently. Patient mix should be considered when interpreting the results of prognostic models, as this may affect results. The addition of a single physiologic variable to administrative models may improve prognostic ability and aid in comparing the quality of care across institutions.

### Ethics, consent, and permissions

Approval for this study was obtained from the UCSF Institutional Review Board, and the requirement for written informed consent was waived (study number 11-07544).
